# No Indication for Routine Resection of Surgical Scars during Cytoreductive Surgery and HIPEC

**DOI:** 10.3390/cancers16112099

**Published:** 2024-05-31

**Authors:** Malin Enblad, Lana Ghanipour, Peter Cashin, Helgi Birgisson, Wilhelm Graf

**Affiliations:** Department of Surgical Sciences, Colorectal Surgery, Uppsala University, 751 85 Uppsala, Sweden

**Keywords:** scar metastases, peritoneal metastases, cytoreductive surgery, HIPEC, pseudomyxoma peritonei, colorectal cancer

## Abstract

**Simple Summary:**

Routine resection of surgical scars could prevent scar recurrences after cytoreductive surgery and hyperthermic intraperitoneal chemotherapy for colorectal peritoneal metastases and pseudomyxoma peritonei. However, there is no clear evidence for resecting all surgical scars, irrespective of macroscopic suspicion of scar metastases, and scar resection is associated with wound complications. Careful macroscopic assessment of surgical scars is needed to avoid routine scar resection. This study aimed to analyze the correlation between macroscopically suspected and microscopically confirmed scar metastases, and to analyze the prognostic impact of not undergoing routine scar resection. This study showed that occult scar metastases were uncommon and patients not undergoing routine scar resection did not have worse recurrence-free or overall survival compared with those undergoing scar resection. Therefore, macroscopically benign-appearing scars can be left without resection, though resection should be performed in case of uncertainty.

**Abstract:**

Background: Careful macroscopic assessment of surgical scars is needed to avoid routine scar resection during cytoreductive surgery (CRS) for peritoneal metastases (PM). This study aimed to analyze the correlation between macroscopically suspected and microscopically confirmed scar metastases (SMs), and to analyze the prognostic impact of not undergoing routine scar resection. Method: All patients with previous surgery, treated with CRS and hyperthermic intraperitoneal chemotherapy, for colorectal PM or pseudomyxoma peritonei (PMP), at Uppsala University Hospital in 2013–2021, were included. Macroscopic SMs in surgical reports were compared with histopathological analyses. Results: In total, 227 patients were included. Among colorectal PM patients (n = 156), SM was macroscopically suspected in 41 (26%) patients, and 63 (40%) underwent scar resection. SM was confirmed in 19 (30%). Among patients with macroscopic suspicion, 45% had confirmed SM (positive predictive value, PPV). A total of 1 of 23 (4%) patients with no macroscopic suspicion had SM (negative predictive value, NPV = 96%). Among the PMP patients (n = 71), SM was macroscopically suspected in 13 (18%), and 28 (39%) underwent scar resection, of whom 12 (43%) had SM. The PPV was 77%. Occult SM was found in 1 of 14 (NPV = 93%). Not undergoing routine scar resection did not affect recurrence-free survival (RFS, *p =* 0.2) or overall survival (OS, *p =* 0.1) in colorectal PM patients or PMP patients (RFS *p =* 0.7, OS *p =* 0.7). Conclusion: Occult SM is uncommon and scar resection does not affect RFS or OS. Therefore, macroscopically benign-appearing scars can be left without resection, though resection should be performed upon suspicion or uncertainty.

## 1. Introduction

Colorectal peritoneal metastases (PM) and pseudomyxoma peritonei (PMP) can be treated successfully with a combination of cytoreductive surgery (CRS) and hyperthermic intraperitoneal chemotherapy (HIPEC), if complete cytoreduction can be achieved [1,2,3]. CRS involves resection of all organs and peritoneal surfaces with macroscopic tumor growth. In addition, the greater omentum is often routinely resected [4], and routine salpingo-oophorectomy is common in postmenopausal women with PMP [5]. There is, however, no clear evidence that surgical scars should be routinely resected. Both colorectal cancer and PMP may lead to the deposition of cells at wound surfaces [6,7], but the prevalence and impact of wound or scar metastases (SMs) are unknown [7,8]. Routine scar resection in patients with previous primary tumor surgery or exploratory laparoscopy/laparotomy for PM could therefore prevent early scar recurrence. 

However, resecting port-site or laparotomy scars with surrounding abdominal wall is not without consequences. Wound complications are particularly common for port-sites off the midline incision [9]. Therefore, diagnostic laparoscopy for peritoneal tumor burden is recommended in Sweden, by using midline port placement if possible. In addition to port-site-associated complications, there are often wound complications related to mesh for reconstruction of larger defects of the abdominal wall [9,10]. Furthermore, scar resection results in large, new wound surfaces where neoplastic cells can attach. Impaired wound healing and infectious complications can also delay recovery and oncologic treatment.

To avoid routine resection of scars, surgeons need to be able to adequately assess the macroscopic appearance of scars. The primary aim of this study was to investigate the correlation between macroscopically suspected and microscopically confirmed SM in patients with colorectal PM or PMP. The secondary aim was to analyze the prognostic impact of not undergoing routine scar resection as part of CRS.

## 2. Materials and Methods

### 2.1. Study Population

All patients treated with initial CRS and HIPEC for confirmed colorectal PM or PMP at Uppsala University Hospital, a national center for surgical treatment of peritoneal surface malignancies, between 2013 and 2021, were eligible for inclusion. Patients with other primary tumors (small bowel adenocarcinoma, mesothelioma, or ovarian cancer), open close/debulking surgery, repeated CRS, patients with no macroscopic PM at exploration, and patients not receiving HIPEC were excluded. Lastly, patients without previous surgery, either primary tumor (colorectal or appendiceal) resection or explorative laparoscopy/laparotomy before CRS and HIPEC, were also excluded. Exploration could be planned diagnostic surgery for peritoneal tumor burden assessment or for other causes, resulting in the discovery of previously unknown PM. Baseline clinical data were retrieved from medical records and radiology and histopathological reports. This study was approved by the regional ethics committee (reference number 2013/203). 

### 2.2. Macroscopic Assessment

The macroscopic assessment of SM was based on descriptions in the surgical reports. No key phrases were used but the five surgeons’ surgical reports followed a template starting with the description of the peritoneal tumor extension and distribution of PM. Accordingly, the tumor extension was assessed routinely at the beginning of surgery, starting systematically with the assessment of the midline scare (if present), followed by an assessment of all abdominal regions (including abdominal wall and other surgical scars) according to the peritoneal cancer index (PCI) [11]. The PCI is calculated by summing lesion size scores (range 0–3) in 13 different abdominal regions. It ranges between 1 and 39. The assessment aims to evaluate if a patient is eligible for CRS and HIPEC. High PCI scores in colorectal PM, PM involving the liver hilum, large vessels, or extensive small bowel involvement, are common reasons for not continuing with planned CRS and HIPEC, as complete cytoreduction is not possible. A completeness of cytoreduction score (CCS, range 0–3) [11] of 0 is pursued in colorectal PM, whereas CCS 0–1 is considered acceptable in PMP. In the present study, an uncertain CCS scoring, ‘0–1’, was arbitrarily classified as 1. 

### 2.3. CRS and HIPEC

CRS was performed by resecting all disease-affected organs and peritoneal surfaces [4]. Resection of surgical scars was not routinely performed. Resections were mainly carried out upon suspicion of SM. In cases of keloid formation or increased consistency of the scar and surrounding tissue, resections were performed not to risk leaving SM. In addition, patients with high PCI or nearby tumor growth sometimes underwent scar resections because of an estimated high risk. HIPEC was performed using the open Coliseum technique [12,13] at the start of the study period and using the closed technique after 2018 [13]. The intraperitoneal agents were most often oxaliplatin and/or irinotecan in colorectal PM patients, and mitomycin C in PMP patients. Perfusion time was 30 min for oxaliplatin/irinotecan and 90 min for Mitomycin C.

### 2.4. Microscopic Assessment

Surgical specimens were fixed in 4% buffered formaldehyde and embedded in paraffin, sliced into 4 µm sections, and stained with hematoxylin and eosin. The primary tumors and the PM were classified in accordance with the World Health Organization’s classification system for tumors of the digestive system [14]. Appendiceal tumors included low-grade appendiceal mucinous neoplasm, high-grade appendiceal mucinous neoplasm, mucinous adenocarcinoma, mucinous signet ring adenocarcinoma, and adenocarcinoma. Colorectal primary tumors were classified as adenocarcinoma, mucinous adenocarcinoma, or signet ring cell adenocarcinoma. For the purpose of this study, patients were divided into colorectal PM and PMP groups. The colorectal PM group included all patients with colorectal primary tumors. The colorectal PM histopathology was described as the primary tumor. The PMP group included all patients with appendiceal PM except those with non-mucinous adenocarcinoma and non-mucinous PM. These patients were included in the colorectal PM group because of similar biological behavior. In Sweden, the Ronnett classification of PMP [15] is primarily used, but for this study, the Peritoneal Surface Oncology Group International (PSOGI) classification was used [16]. The PSOGI classification consists of three mucinous carcinoma peritonei (MCP) grades, G1–G3. Patients with acellular mucin were reported separately [16].

### 2.5. Statistical Analysis

Continuous data are presented as medians with interquartile ranges (IQRs). Fisher’s exact test was used for comparisons of categorical data and the Mann–Whitney U test was used to compare continuous data. Positive predictive value (PPV), negative predictive value (NPV), sensitivity, and specificity were used to analyze the ability of surgeons to assess scars for metastases. Overall survival (OS) and recurrence-free survival (RFS) were calculated using Kaplan–Meier curves and the cumulative proportions surviving/recurring within five years after CRS are presented with 95% confidence intervals (CIs). Patients were censored at the last follow-up. Follow-up data were retrieved on 5 October 2022. The log-rank test was used to compare differences in OS and RFS. Risk factors for recurrence and death were analyzed with univariate and multivariate Cox proportional hazard regression analyses and presented as hazard ratios (HRs) with 95% CIs. A *p*-value < 0.05 was considered statistically significant. R version 4.2.2 (R foundation for Statistical Computing, Vienna, Austria) was used for statistical analyses.

## 3. Results

### 3.1. Study Population

After excluding patients with other primary tumors (n = 35), open close/debulking surgery (n = 74), repeated CRS (n = 18), patients with no macroscopic PM at exploration, and patients not receiving HIPEC (n = 72), 303 patients were eligible for analysis. Of these, 76 patients with synchronous PM had not undergone exploratory laparoscopy/laparotomy before CRS and HIPEC and were excluded. This left 227 patients for final analysis of SM. Patients were divided into groups with colorectal PM (n = 156) or PMP (n = 71) and these groups were analyzed separately. The baseline clinical characteristics of the patients are shown in Table 1. All but one patient with PMP had synchronous disease and underwent explorative laparoscopy/laparotomy before CRS and HIPEC. Primary tumor resection was the only previous surgery in 46 (29%) of the colorectal PM patients, whereas the remaining portion underwent exploration before CRS and HIPEC. In one patient categorized as PMP, the primary tumor could not be clearly identified because of extensive mucinous tumor growth.

The 76 excluded patients with synchronous PM without explorative laparoscopy/laparotomy encompassed 54 PMP patients and 22 colorectal PM patients. The excluded PMP patients had high PCI scores (median 26, IQR 13–33) and typical radiology and clinical presentation. The excluded colorectal PM patients had a median PCI score of 12 (IQR 5–17) and varying primary tumor localization (right-sided = 11) and histopathology (mucinous/signet ring = 12).

### 3.2. Macroscopic Assessment

**Figure 1:**
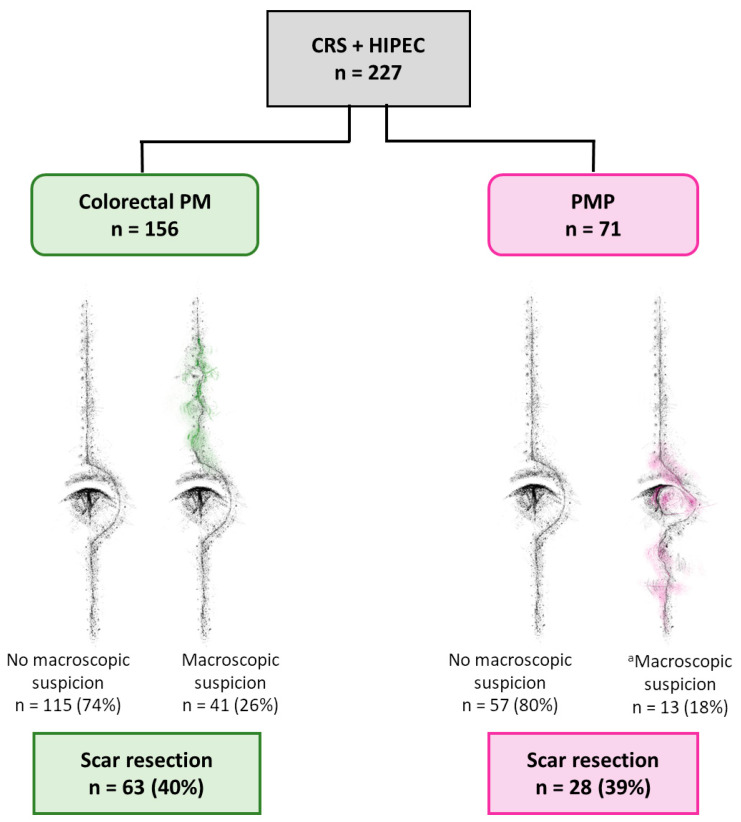
Frequency of macroscopic suspicion of scar metastases and scar resection in patients with colorectal peritoneal metastases (PM) or pseudomyxoma peritonei (PMP), treated with cytoreductive surgery (CRS) and hyperthermic intraperitoneal chemotherapy (HIPEC). ^a^ For one patient, the surgeon clearly wrote that there was uncertainty regarding whether there was macroscopic suspicion or not. This patient could not be categorized as macroscopic suspicion or no macroscopic suspicion.

The surgeon suspected SM in 41 (26%) and 13 (18%) of the colorectal PM and PMP patients, respectively. In one PMP patient, the surgeon expressed that it was difficult to judge if there was an SM or not. Scar resection was performed in 40% of the colorectal PM patients and 39% of the PMP patients (Figure 1). 

### 3.3. Histopathology of Colorectal PM

**Figure 2:**
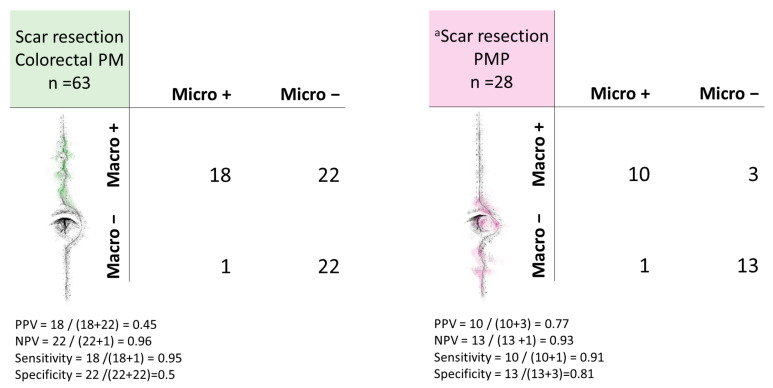
Cross-tabulations of macroscopic suspicion of scar metastases versus microscopic results in patients with colorectal peritoneal metastases (PM) or pseudomyxoma peritonei (PMP). ^a^ For one patient, the surgeon clearly wrote that there was uncertainty regarding whether there was macroscopic suspicion or not. This patient could not be categorized as Micro+ or Macro−, and excluded from the cross-tabulations. This patient had microscopically confirmed scar metastasis. PPV; positive predictive value, NPV; negative predictive value, Macro; macroscopic suspicion, Micro; histopathological result.

A total of 19 (30%) of the patients with colorectal PM who underwent scar resection had microscopically confirmed SM. The surgeon’s macroscopic suspicion of SM was confirmed in 18 of 40 (45%) patients, corresponding to the PPV. Occult SM was present in 1 of 23 (4%) patients. Hence, the surgeon did not suspect SM, but the scar was still resected (NPV = 96%). This patient had synchronous PM of adenocarcinoma and had a PCI of 10. The sensitivity of the macroscopic assessment was calculated to 95% and the specificity to 50% (Figure 2). 

Table 2 shows the clinical and histopathological characteristics of colorectal PM patients with and without SM. There were no significant differences. Three patients without SM had no evidence of PM, PCI < 5, and had not received neoadjuvant treatment. The histopathologically confirmed SM were localized in midline laparotomy scars (n = 14), port-site scars (n = 4), and Phannenstiel incision scars (n = 2). 

### 3.4. Histopathology of PMP

In total, 12 of 28 (43%) patients with scar resection had microscopically confirmed SM. In the one patient where the surgeon expressed that there was uncertainty regarding the macroscopic appearance, neoplastic cells were confirmed. The remaining patients had available information on both macroscopic suspicion and histopathological results. These are shown in Figure 2. SM was confirmed in 10 of 13 (77%) patients with macroscopic suspicion, yielding the PPV. One patient was incorrectly judged as not having SM and the NPV was calculated to 93%. This patient had a PCI score 19 and PSOGI MCP G1 in the other surgical specimens, but only acellular mucin in the scar. The sensitivity and specificity were 91% and 81%, respectively.

The clinical and histopathological characteristics of patients with and without SM are found in Table 3. The only significant difference was age (*p*-value 0.03). In six (50%) of the patients with SM, no neoplastic cells were found in the scar, but acellular mucin was. In three of these patients, the remaining surgical specimens also contained only acellular mucin. In the other three, PSOGI MCP G1/2 were found in other specimens. The histopathologically confirmed SMs were localized in midline laparotomy scars (n = 4), port-site scars (n = 6), and McBurney incision scars (n = 2).

### 3.5. Recurrence and Survival

**Figure 3:**
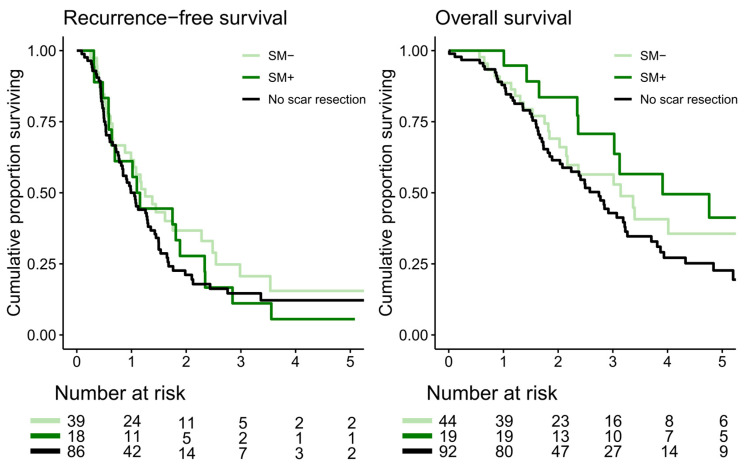
Recurrence-free and overall survival for patients with colorectal peritoneal metastases, with or without scar metastasis (SM+ vs. SM−), and patients with no scar resection. Recurrence-free and overall survival for patients with no scar resection were compared with all patients undergoing scar resection (SM+ and SM−).

In colorectal PM patients, there was no difference in 5-year RFS between those with confirmed SM (6%, 95% CI 1–37) and those without SM (15%, 95% CI 6–39, *p* = 0.5). Patients who did not undergo resection had a 5-year RFS of 12% (95% CI 6–24), which was not worse than that of patients who underwent scar resection (*p* = 0.2). Recurrence information was missing in thirteen colorectal PM patients, who were excluded from the RFS analysis. Five-year OS in patients with confirmed SM (41%, 95% CI 22–77) was compared with that of patients without SM (36%, 95% CI 22–59). There was no difference in survival (*p* = 0.6). Patients who did not undergo resection had a 5-year OS of 23% (95% CI 14–36), which was not worse compared with that of patients who underwent scar resection (*p* = 0.1, Figure 3). One patient was lost to follow-up due to emigration and excluded from the OS analysis.

**Figure 4:**
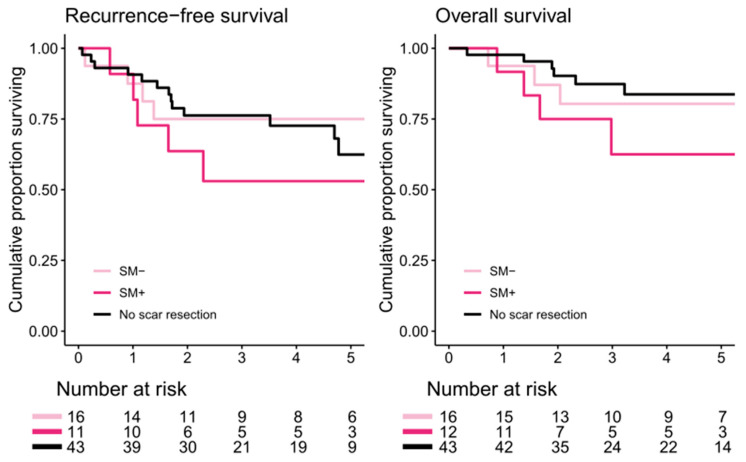
Recurrence-free and overall survival for patients with pseudomyxoma peritonei, with or without scar metastasis (SM+ vs. SM−), and patients with no scar resection. Recurrence-free and overall survival for patients with no scar resection were compared with all patients undergoing scar resection (SM+ and SM−).

In PMP patients, there was no difference in 5-year RFS in those who had confirmed SM (53%; 95% CI 30–94) compared with in those without SM (75%; 95% CI 57–100, *p* = 0.3). Patients without scar resection had a 5-year RFS of 62% (95% CI 47–83). There was no difference in RFS between those who underwent scar resection and those who did not (*p* = 0.7). In one patient, recurrence information was missing. This patient was not included in the RFS analysis. Five-year OS in patients with SM was 63% (95% CI 39–100) and that in those without SM was 80% (95% CI 63–100; *p* = 0.4). Patients without scar resection had a 5-year OS of 84% (95% CI 72–97). There were no differences in survival rates between those who underwent scar resection and those who did not (*p* = 0.7, Figure 4).

The impacts on RFS and OS of not undergoing scar resection were analyzed with multivariate Cox regression analysis with the variables PCI and CCS. Not undergoing scar resection was not a risk factor for recurrence or death in colorectal PM (Table 4) or PMP (Table 5).

## 4. Discussion

This study shows that SMs are recognized macroscopically by surgeons at the beginning of CRS and that occult SMs are uncommon. At our Swedish center for peritoneal surface malignancies, scar resection has not been carried out routinely, and this study shows that neither RFS nor OS were worse in patients without scar resection.

The prevalence of histopathologically confirmed SM among patients undergoing scar resection was 30% in colorectal PM patients and 43% in PMP patients (35% in total). The majority had metastases in the midline laparotomy scar. The prevalence of SM in patients with colorectal PM or PMP undergoing CRS has not been the focus of other studies, but similar prevalence rates have been reported for mixed primary tumor origins (appendiceal, colorectal, small bowel, gastric, mesothelioma, gynecological) [9]. On the subject of diagnostic laparoscopy for peritoneal tumor burden assessment, the prevalence of port-site metastases in a smaller study of mixed primary tumors was 34% [17]. In a larger study, the prevalence was only 0.4% [18]. PMP recurrence often involves the CRS midline laparotomy scar [7]. In this study, the majority of patients undergoing scar resection had a previous surgery with manifest PM, especially in PMP, where only one patient had metachronous PM and had undergone primary tumor surgery only. In colorectal PM patients undergoing scar resection, 46 (29%) had metachronous PM and previous primary tumor surgery only. Of these, only four patients had confirmed SM, indicating that SM is uncommon in patients who have not been operated on with manifest PM.

The question is whether the prevalence itself justifies scar resection in all patients, irrespective of macroscopic suspicion. This study shows that occult SM was found in one (4%) and one (7%) of colorectal PM and PMP patients, respectively. Higher prevalence of occult SM was found in one study of mixed primary tumors [9]. In another study, it was found that resections often resulted in a benign outcome [19]. This was seen also in the present study, especially for colorectal PM, where 50% of the suspected SMs were benign. Benign lesions, specimens from CRS without neoplastic cells despite suspicion, have been described previously, as has acellular mucin [20]. Acellular mucin has received increased interest and should be separated from PSOGI MCP G1 [16]. The one patient with PMP and occult SM had acellular mucin only in the scar. Acellular mucin is sometimes the only sign of peritoneal disease. It is associated with excellent prognosis, although neoplastic cells are sometimes found upon re-examination [21]. Based on these results, routine resections of all surgical scars in all patients are not justified. With that being said, many scars resected during the study period were without suspicion, but often resected because of keloid formation, large peritoneal tumor burden, or in case of preoperative radiological suspicion. Such cases would be difficult to leave without resection.

The prevalence of occult SM in patients not undergoing scar resection (60% of both colorectal PM and PMP patients) is unknown. However, these patients had comparable RFS and OS to those who underwent scar resection. Notably, the OS curve for colorectal PM patients not undergoing scar resection showed a tendency for poorer survival in these patients. The multivariate Cox regression analysis did not confirm this. This further strengthens the idea that macroscopically benign-looking scars can be left without resection and that CRS is all about weighing the extent of surgical resections against the risks of postoperative complications, morbidity and recurrence. 

This study has some noteworthy limitations. First, the macroscopic assessments were retrospectively collected from surgical reports, causing a risk of misinterpretation. Of note, the reasons for scar resections in cases of no suspicion of SM were not clearly described. In addition, scar resection was not performed routinely, meaning that the exact prevalence of occult SM was unknown. On the other hand, this allowed for analysis of survival differences between patients with and without resection. Third, the exact sites of recurrence after CRS and HIPEC were unknown and it would have been interesting to see if the prevalence of scar recurrence differed between patients with and without resection. However, one described risk factor for scar recurrence after CRS is gross SM resected during CRS. Thus, scar resection of SM is in itself a risk factor for SM [7]. 

## 5. Conclusions

Occult SMs are uncommon and macroscopic suspicion is often microscopically benign. Patients not undergoing routine scar resection did not have worse RFS or OS compared with those undergoing scar resection. Therefore, there is no strong indication for routine resection of all surgical scars during CRS, and macroscopically benign-appearing scars can be left without resection. However, resection should be performed upon uncertainty, and—needless to say—in case of suspected involvement.

## Figures and Tables

**Table 1:** Baseline clinical characteristics of patients with colorectal peritoneal metastases or pseudomyxoma peritonei treated with cytoreductive surgery and hyperthermic intraperitoneal chemotherapy, 2013–2021.

Characteristics	Colorectal PM n = 156 n (%)	PMP n = 71 n (%)
**#Sex**		
Male	71 (46)	28 (39)
Female	85 (54)	43 (61)
**Age, years (median, IQR)**	67 (56–71)	59 (51–68)
**Primary tumor localization**		
Appendix	6 (4)	70 (99)
Right colon	73 (47)	0 (0)
Left colon	52 (33)	0 (0)
Rectum	20 (13)	0 (0)
Colorectal NOS	5 (3)	1 (1)
**Previous surgery**		
^a^ Primary tumor resection	46 (29)	1 (1)
^b^ Exploratory laparoscopy/laparotomy	85 (54)	68 (96)
^c^ Both	25 (16)	1 (1)
**Neoadjuvant treatment**	37 (24)	2 (3)
**PCI (median, IQR)**	10 (5–18)	9 (6–20)
**CCS**		
0	149 (96)	60 (85)
1	7 (4)	11 (15)

^a^ Resection of a primary tumor with curative intent followed by metachronous PM. ^b^ Explorative laparoscopy or laparotomy for synchronous PM. ^c^ Resection of a primary tumor with curative intent and later laparoscopy or laparotomy for metachronous PM. PMP; pseudomyxoma peritonei, PM; peritoneal metastases, IQR; interquartile range, NOS; not otherwise specified, PCI; peritoneal cancer index, CCS; completeness of cytoreduction score.

**Table 2:** Clinical and histopathological characteristics of patients with colorectal peritoneal metastases, with or without confirmed scar metastases.

Characteristics	Scar Metastasis n = 19 n (%)	No Scar Metastasis n = 44 n (%)	*p*-Value
**Sex**			*0.58*
Male	10 (53)	19 (43)	
Female	9 (47)	25 (57)	
**Age, years (median, IQR)**	67 (59–71)	65 (53–71)	*0.52*
**Previous surgery**			*0.10*
^a^ Primary tumor resection	4 (21)	2 (5)	
^b^ Exploratory laparoscopy/laparotomy	10 (53)	31 (70)	
^c^ Both	5 (26)	11 (25)	
**Primary tumor localization**			*0.73*
Appendix	0 (0)	3 (7)	
Right colon	9 (47)	23 (52)	
Left colon	6 (32)	11 (25)	
Rectum	4 (21)	7 (16)	
Colorectal NOS	0 (0)	0 (0)	
**^d^ Peritoneal metastasis histopathology**			*0.27*
Adenocarcinoma	10 (53)	6 (14)	
Mucinous adenocarcinoma	7 (37)	12 (27)	
Signet ring cell carcinoma	2 (11)	1 (2)	
No neoplastic disease	0 (0)	3 (7)	
**PCI (median, IQR)**	12 (9–18)	10 (6–17)	*0.33*
**CCS**			*1.00*
0	19 (100)	42 (95)	
1	0 (0)	2 (5)	

^a^ Resection of a primary tumor with curative intent, followed by metachronous PM. ^b^ Explorative laparoscopy or laparotomy for synchronous PM. ^c^ Resection of a primary tumor with curative intent and later laparoscopy or laparotomy for metachronous PM. ^d^ Peritoneal metastasis histopathology describes the dominating histopathology of the PM. IQR; interquartile range, PM; peritoneal metastases, PCI; peritoneal cancer index, CCS; completeness of cytoreduction score.

**Table 3:** Clinical and histopathological characteristics of patients with pseudomyxoma peritonei, with or without confirmed scar metastases.

Characteristics	Scar Metastasis n = 12 n (%)	No Scar Metastasis n = 16 n (%)	*p*-Value
**Sex**			*0.70*
Male	6 (50)	6 (38)	
Female	6 (50)	10 (63)	
**Age, years (median, IQR)**	67 (55–72)	52 (46–57)	*0.03*
**Previous surgery**			*0.43*
^a^ Primary tumor resection	0 (0)	0 (0)	
^b^ Exploratory laparoscopy/laparotomy	11 (92)	16 (100)	
^c^ Both	1 (8)	0 (0)	
**^d^ Peritoneal metastasis histopathology**			*0.40*
Acellular mucin	3 (25)	2 (13)	
PSOGI MCP G1	1 (8)	4 (25)	
PSOGI MCP G2	5 (45)	5 (31)	
PSOGI MCP G3	3 (25)	2 (13)	
No neoplastic disease	0 (0)	3 (19)	
**PCI (median, IQR)**	20 (11–28)	12 (6–22)	*0.77*
**CCS**			*0.65*
0	9 (75)	12 (75)	
1	3 (25)	4 (25)	

^a^ Resection of a primary tumor with curative intent followed by metachronous PM. ^b^ Explorative laparoscopy or laparotomy for synchronous PM. ^c^ Resection of a primary tumor with curative intent and later laparoscopy or laparotomy for metachronous PM. ^d^ Peritoneal metastasis histopathology describes the dominating histopathology of the PM. IQR; interquartile range, PM; peritoneal metastases, PSOGI; Peritoneal Surface Oncology Group International, MCP; mucinous carcinoma peritonei, PCI; peritoneal cancer index, CCS; completeness of cytoreduction score.

**Table 4:** Univariate and multivariate Cox proportional regression hazard analysis for risk of recurrence and death in patients with colorectal peritoneal metastases treated with cytoreductive surgery and hyperthermic intraperitoneal chemotherapy.

	RFS Univariate HR (95% CI)	RFS Multivariate HR (95% CI)	OS Univariate HR (95% CI)	OS Multivariate HR (95% CI)
**PCI**	**1.04 (1.01–1.06)**	**1.05 (1.02–1.07)**	**1.06 (1.03–1.07)**	**1.06 (1.03–1.09)**
**CCS**				
0	1.00	1.00	1.00	1.00
1	1.27 (0.51–3.13)	0.48 (0.17–1.32)	2.04 (0.82–5.03)	0.63 (0.23–1.71)
**Scar resection**				
No	1.00	1.00	1.00	1.00
Yes	0.80 (0.55–1.16)	0.69 (0.47–1.01)	0.71 (0.47–1.09)	0.67 (0.44–1.04)

RFS; recurrence-free survival, OS; overall survival, HR; hazard ratio, CI; confidence interval, PCI; peritoneal cancer index, CCS; completeness of cytoreduction score.

**Table 5:** Univariate and multivariate Cox proportional regression hazard analysis for risk of recurrence and death in patients with pseudomyxoma peritonei treated with cytoreductive surgery and hyperthermic intraperitoneal chemotherapy.

	RFS Univariate HR (95% CI)	RFS Multivariate HR (95% CI)	OS Univariate HR (95% CI)	OS Multivariate HR (95% CI)
**PCI**	**1.06 (1.02–1.11)**	**1.011(1.05–1.18**	1.04 (0.99–1.09)	**1.06 (1.03–1.09)**
**CCS**				
0	1.00	1.00	1.00	1.00
1	0.80 (0.24–2.73)	**0.18 (0.04–0.74)**	0.51 (0.11–2.82)	**0.16 (0.03–0.90)**
**Scar resection**				
No	1.00	1.00	1.00	1.00
Yes	1.16 (0.49–2.71)	0.86 (0.34–2.15)	1.09 (0.41–2.87)	1.09 (1.02–1.16)

RFS; recurrence-free survival, OS; overall survival, HR; hazard ratio, CI; confidence interval, PCI; peritoneal cancer index, CCS; completeness of cytoreduction score.

## Data Availability

The data presented in this study are not publicly available, in accordance with requirements from the ethics committee, as the data contain personal information.

## References

[1] Verwaal V.J., Bruin S., Boot H., van Slooten G., van Tinteren H. (2008). 8-year follow-up of randomized trial: Cytoreduction and hyperthermic intraperitoneal chemotherapy versus systemic chemotherapy in patients with peritoneal carcinomatosis of colorectal cancer. Ann. Surg. Oncol..

[2] Huang C.Q., Min Y., Wang S.Y., Yang X.J., Liu Y., Xiong B., Yonemura Y., Li Y. (2017). Cytoreductive surgery plus hyperthermic intraperitoneal chemotherapy improves survival for peritoneal carcinomatosis from colorectal cancer: A systematic review and meta-analysis of current evidence. Oncotarget.

[3] Chua T.C., Moran B.J., Sugarbaker P.H., Levine E.A., Glehen O., Gilly F.N., Baratti D., Deraco M., Elias D., Sardi A. (2012). Early- and long-term outcome data of patients with pseudomyxoma peritonei from appendiceal origin treated by a strategy of cytoreductive surgery and hyperthermic intraperitoneal chemotherapy. J. Clin. Oncol..

[4] Sugarbaker P.H. (1995). Peritonectomy procedures. Ann. Surg..

[5] Mittal R., Chandramohan A., Moran B. (2017). Pseudomyxoma peritonei: Natural history and treatment. Int. J. Hyperthermia.

[6] Reilly W.T., Nelson H., Schroeder G., Wieand H.S., Bolton J., O‘Connell M.J. (1996). Wound recurrence following conventional treatment of colorectal cancer. A rare but perhaps underestimated problem. Dis. Colon. Rectum..

[7] Zoetmulder F.A., Sugarbaker P.H. (1996). Patterns of failure following treatment of pseudomyxoma peritonei of appendiceal origin. Eur. J. Cancer..

[8] Curet M.J. (2004). Port site metastases. Am. J. Surg..

[9] Nunez M.F., Sardi A., Nieroda C., Jimenez W., Sittig M., MacDonald R., Aydin N., Milovanov V., Gushchin V. (2015). Morbidity of the abdominal wall resection and reconstruction after cytoreductive surgery and hyperthermic intraperitoneal chemotherapy (CRS/HIPEC). Ann. Surg. Oncol..

[10] Parikh R., Shah S., Dhurandhar V., Alzahrani N., Fisher O.M., Arrowaili A., Liauw W., Morris D. (2019). An analysis of the morbidity associated with abdominal wall resection and reconstruction after cytoreductive surgery and hyperthermic intraperitoneal chemotherapy (CRS/HIPEC). Eur. J. Surg. Oncol..

[11] Jacquet P., Sugarbaker P.H. (1996). Clinical research methodologies in diagnosis and staging of patients with peritoneal carcinomatosis. Cancer Treat. Res..

[12] Stephens A.D., Alderman R., Chang D., Edwards G.D., Esquivel J., Sebbag G., Steves M.A., Sugarbaker P.H. (1999). Morbidity and mortality analysis of 200 treatments with cytoreductive surgery and hyperthermic intraoperative intraperitoneal chemotherapy using the coliseum technique. Ann. Surg. Oncol..

[13] Gonzalez-Moreno S., Gonzalez-Bayon L.A., Ortega-Perez G. (2010). Hyperthermic intraperitoneal chemotherapy: Rationale and technique. World J. Gastrointest. Oncol..

[14] Bosman F., Carniero F., Hruban R., Theise N. (2010). WHO Classification of Tumours of the Digestive System.

[15] Ronnett B.M., Zahn C.M., Kurman R.J., Kass M.E., Sugarbaker P.H., Shmookler B.M. (1995). Disseminated peritoneal adenomucinosis and peritoneal mucinous carcinomatosis. A clinicopathologic analysis of 109 cases with emphasis on distinguishing pathologic features, site of origin, prognosis, and relationship to “pseudomyxoma peritonei”. Am. J. Surg. Pathol..

[16] Carr N.J., Cecil T.D., Mohamed F., Sobin L.H., Sugarbaker P.H., Gonzalez-Moreno S., Taflampas P., Chapman S., Moran B.J. (2016). A Consensus for Classification and Pathologic Reporting of Pseudomyxoma Peritonei and Associated Appendiceal Neoplasia: The Results of the Peritoneal Surface Oncology Group International (PSOGI) Modified Delphi Process. Am. J. Surg. Pathol..

[17] Nunez M.F., Sardi A., Jimenez W., Nieroda C., Sittig M., MacDonald R., Aydin N., Milovanov V., Gushchin V. (2015). Port-site metastases is an independent prognostic factor in patients with peritoneal carcinomatosis. Ann. Surg. Oncol..

[18] Carboni F., Federici O., Giofre M., Valle M. (2020). An 18-Year Experience in Diagnostic Laparoscopy of Peritoneal Carcinomatosis: Results from 744 Patients. J. Gastrointest. Surg..

[19] Berger Y., Jacoby H., Kaufmann M.I., Ben-Yaacov A., Westreich G., Sharon I., Barda L., Sharif N., Nadler R., Horesh N. (2019). Correlation Between Intraoperative and Pathological Findings for Patients Undergoing Cytoreductive Surgery and Hyperthermic Intraperitoneal Chemotherapy. Ann. Surg. Oncol..

[20] Enblad M., Birgisson H., Wanders A., Skoldberg F., Ghanipour L., Graf W. (2016). Importance of Absent Neoplastic Epithelium in Patients Treated With Cytoreductive Surgery and Hyperthermic Intraperitoneal Chemotherapy. Ann. Surg. Oncol..

[21] Al-Azzawi M., Misdraji J., van Velthuysen M.F., Shia J., Taggart M.W., Yantiss R.K., Svrcek M., Carr N. (2020). Acellular mucin in pseudomyxoma peritonei of appendiceal origin: What is adequate sampling for histopathology?. J. Clin. Pathol..

